# NIR Light-Triggered Chemo-Phototherapy by ICG Functionalized MWNTs for Synergistic Tumor-Targeted Delivery

**DOI:** 10.3390/pharmaceutics13122145

**Published:** 2021-12-13

**Authors:** Lu Tang, Aining Zhang, Yijun Mei, Qiaqia Xiao, Xiangting Xu, Wei Wang

**Affiliations:** 1 State Key Laboratory of Natural Medicines, Department of Pharmaceutics, School of Pharmacy, China Pharmaceutical University, Nanjing 210009, China; lutang@stu.cpu.edu.cn (L.T.); zaining@stu.cpu.edu.cn (A.Z.); yjmei@stu.cpu.edu.cn (Y.M.); xiaoqiaqia@stu.cpu.edu.cn (Q.X.); xiangtingx_cpu@163.com (X.X.); 2 NMPA Key Laboratory for Research and Evaluation of Pharmaceutical Preparations and Excipients, China Pharmaceutical University, Nanjing 210009, China

**Keywords:** multi-walled carbon nanotube, photothermal therapy, indocyanine green, synergistic strategy, cancer treatment, targeted drug delivery

## Abstract

The combinational application of photothermal therapy (PTT), chemotherapy, and nanotechnology is a booming therapeutic strategy for cancer treatment. Multi-walled carbon nanotube (MWNT) is often utilized as drug carrier in biomedical fields with excellent photothermal properties, and indocyanine green (ICG) is a near-infrared (NIR) dye approved by FDA. In addition, ICG is also a photothermal agent that can strongly absorb light energy for tumor ablation. Herein, we explored a synergistic strategy by connecting MWNT and a kind of ICG derivate ICG-NH_2_ through hyaluronic acid (HA) that possesses CD44 receptor targeting ability, which largely enhanced the PTT effect of both MWNT and ICG-NH_2_. To realize the synergistic therapeutic effect of chemotherapy and phototherapy, doxorubicin (DOX) was attached on the wall of MWNT via π–π interaction to obtain the final MWNT-HA-ICG/DOX nanocomplexes. Both in vitro and in vivo experiments verified the great therapeutic efficacy of MWNT-HA-ICG/DOX nanocomplexes, which was characterized by improved photothermal performance, strengthened cytotoxicity, and elevated tumor growth inhibition based on MCF-7 tumor models. Therefore, this synergistic strategy we report here might offer a new idea with promising application prospect for cancer treatment.

## 1. Introduction

Cancer remains one of the deadly diseases that seriously threatens human health. Despite the encouraging progress of medical advancement, effective therapeutic methods against cancer are still insufficient. Conventional treatment modalities such as chemotherapy, radiotherapy, and surgery often undergo many drawbacks such as unavoidable side effects, severe pain, potential development of drug resistance, and inadequate effectiveness due to the instability and rapid clearance of drugs, which all cause unsatisfactory outcomes of anticancer therapy [1,2,3]. To overcome the limitations mentioned above, more and more attempts based on targeted drug delivery have been developed to improve cancer treatment efficacy. In this context, nanomaterial-mediated platforms have been widely explored in anticancer drug delivery, which serve as effective carriers of both therapeutic agents and diagnostic agents due to their distinctive properties and unique advantages, such as increased drug stability, reduced systemic toxicity, improved pharmacokinetics, elevated bioavailability, precise drug transportation capability, and controlled drug release ability [4,5,6,7].

From all the nanomaterials, carbon nanotubes (CNTs) have captured many researchers’ attention due to their multiple application possibilities in cancer theranostics [8]. CNTs possess tiny tubular shapes composed of carbon atoms that are ordered to form a honeycomb nanostructure with many unique physicochemical characteristics [9]. Generally, CNTs can be sorted into either single-wall carbon nanotubes (SWNTs) or multi-wall carbon nanotubes (MWNTs) according to the sheet number of carbon atoms, both playing a significant role in anticancer therapy [10]. Due to their multifunctionality, CNTs are widely investigated in cancer treatment through various therapeutic modalities. For instance, the strong absorption of CNTs in near-infrared (NIR) regions make them ideal candidates in phototherapy. In addition, CNTs can transform the laser energy to acoustic signals and display excellent resonant Raman scattering and photoluminescence in NIR region, which are all advantageous to their application in cancer imaging [11]. Moreover, many studies have reported that CNTs can be taken up by various cell types due to their needle-like architecture, which enhances their deep tumor penetration to act as ideal drug delivery platforms in anticancer therapy [12]. Additionally, the ultra-high surface area of CNTs for drug loading also benefits their utilization in anticancer drug delivery. However, there are still some obstacles that limit their broad application in biomedical fields. For example, the unique nanostructure of CNTs promotes their hydrophobicity and causes cytotoxicity [13]. Therefore, functionalizing CNTs to increase their hydrophilicity as well as attenuate their inherent cytotoxicity is a key point to improve their biocompatibility for safe application in cancer therapy [14].

Phototherapy is an emerging therapeutic modality that takes advantage of laser energy to eliminate the target tumor due to its high selectivity [15,16]. Photothermal therapy (PTT) and photodynamic therapy (PDT) are two typical phototherapeutic approaches, and NIR light is always used as the light source for phototherapy due to its deep tissue penetration capability [17]. The conversion of absorbed photon energy into thermal energy to cause hyperthermia for tumor ablation is known as PTT, while the absorbance of specific light energy to produce cytotoxic reactive oxygen species (ROS) is termed as PDT, both of which play critical roles in cancer phototherapy [18]. Moreover, in recent decades, light-triggered therapies have been developed as safe treatment modalities to ablate numerous tumors with great effectiveness [18,19]. PTT, one of the representative phototherapy methods, has gained considerable attention in cancer treatment due to its various merits such as minimal trauma, easy implementation, and fewer side effects, which can effectively inhibit solid tumor growth through localized thermal destruction [20]. Indocyanine green (ICG) is a NIR dye approved by FDA for application in phototherapy due to its strong light absorbance in the NIR window [21,22]. However, some intrinsic limitations such as poor solubility, instability, concentration-dependent aggregation, and rapid clearance largely impede the effectiveness of ICG in phototherapy [23]. With the aim of overcoming these aforementioned drawbacks, construction of an appropriate drug carrier to load ICG is of great necessity [24].

**Scheme 1:**
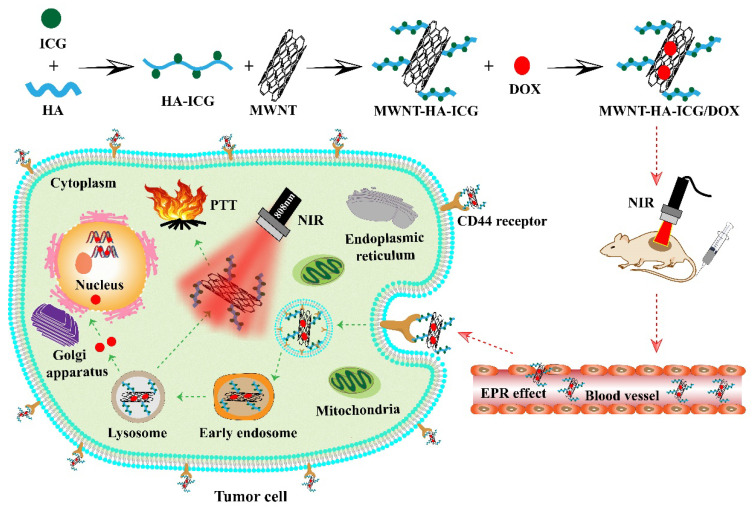
Schematic illustration of the construction of MWNT-HA-ICG/DOX nanocomplexes for synergistic cancer chemo-phototherapy.

In this work, a synergistic chemo-phototherapy was achieved by adopting MWNT to carry photothermal agent ICG-NH_2_ and anticancer drug doxorubicin (DOX) (Scheme 1). Briefly, ICG-NH_2_ was conjugated with hyaluronic acid (HA) by an amide bond to form the HA-ICG conjugate, which improved the water solubility of ICG-NH_2_. Then, MWNT-HA-ICG was synthesized via an ester bond between the carboxyl groups on acidified MWNT and hydroxyl groups on HA, which significantly enhanced the photothermal performance compared to MWNT or ICG-NH_2_ alone. In addition, the connection through HA could not only elevate the targetability of MWNT-HA-ICG due to its affinity with CD44 receptors that are over-expressed on the membrane of many tumor cells, but also endow the whole drug delivery system with good dispersity and biocompatibility [25,26,27]. Furthermore, to realize synergistic chemo-photothermal therapy, DOX was attached on the surface of MWNT by a non-covalent π–π bond to obtain the final MWNT-HA-ICG/DOX nanocomplexes (MWNT-HA-ICG/DOX). The targeted delivery of DOX through MWNT-HA-ICG greatly enhanced the therapeutic efficacy of DOX compared to free administration, while causing reduced side effects due to its non-specificity [28,29]. The novelty of this work is that we combined the drug carrier MWNT with good optical property and photothermal agent ICG-NH_2_ in one platform to achieve synergistic photothermal therapeutic effect. Meanwhile, HA was used a targeting ligand in this nanosystem, which not only increased the targetability of the whole delivery platform, but also elevated the overall biocompability. To further improve the therapeutic efficacy, the classical anticancer drug DOX was employed in the constructed delivery system to take chemotherapy effect. Altogether, this integrated nanoplatform using combined chemo-phototherapy provides a promising strategy for precise cancer therapy.

## 2. Materials and Methods

### 2.1. Materials, Cell Lines and Animals

MWNTs (diameter: 10–20 nm, length: 5–15 μm) were purchased from Shenzhen Nanotechnologies Port Co., Ltd. (Shenzhen, China). DOX·HCl was purchased from Nanjing Chemlin Chemical Industry Co., Ltd. (Nanjing, China). ICG-NH_2_ was synthesized by Prof. Dun Wang in Shenyang Pharmaceutical University. Sodium hyaluronic acid (MW = 5 kDa), N-(3-dimethylaminopropyl)-N’-ethylcarbodiimide hydrochloride (EDC·HCl), and N-hydroxysuccinimide (NHS) were obtained from Aladdin Reagent Database Inc. (Shanghai, China). Concentrated sulfuric acid (98%), concentrated nitric acid (65%), 4-dimethylaminopyridine (DMAP), and N,N’-Carbonyldiimidazole (CDI) were purchased from Aladdin Reagent Database Inc. (Shanghai, China). 4′, 6-diamidino-2-phenylindole (DAPI) were obtained from Beyotime Institute of Biotechnology (Shanghai, China). All other reagents were of analytical grade and were commercially available.

Human breast cancer cell line MCF-7 were purchased from the Cell Bank of Shanghai Institute of Biochemistry and Cell Biology, Chinese Academy of Sciences (Shanghai, China) and cultured in DMEM medium (Hyclone) supplemented with 10% FBS (Hyclone), 100 U/mL penicillin, and 100 μg/mL streptomycin in a humidified atmosphere of 5% CO_2_ at 37 °C.

Female BALB/c nude mice (4–6 weeks, 18–20 g) were purchased from Qinglongshan Animal Farm (Nanjing, China) and were given free access to food and water. All mice were cared for in compliance with the National Institute of Health (NIH) Guidelines for the Care and Use of Laboratory Animals and was approved by the Ethics Committee of China Pharmaceutical University (Ethics Code: 2021-12-002). The tumor xenograft models were established by injecting MCF-7 cell suspensions (1 × 10^6^ cells) subcutaneously into the right flank region of nude mice. A caliper was used to measure tumor sizes, and the tumor volume was calculated as follows: (tumor length) × (tumor width)^2^/2.

### 2.2. Preparation of MWNT-HA-ICG/DOX Nanocomplexes

#### 2.2.1. Carboxylation of MWNTs

Pristine MWNTs (300 mg) were suspended in mixed acid (H_2_SO_4_/HNO_3_, *v*/*v* = 3:1, 140 mL) by ultrasonication for 18 h. Then, the suspension was diluted with deionized water and filtered through a 0.22 μm micro-porous membrane, followed by repeated washing through deionized water until the pH of filtrate was neutral. The solid product on the membrane was then redispersed in deionized water and lyophilized to obtain the final MWNT-COOH, which was stored at room temperature for further use.

#### 2.2.2. Synthesis of HA-ICG

Firstly, HA (60 mg) and NHS (43.79 mg) were dissolved in the mixture of water and DMF (1:3), after which EDC solution was added into the HA solution and stirred for 1 h to activate the carboxyl groups of HA. Then, ICG-NH_2_ (44.4 mg) was dissolved in the mixture of water and DMF (1:3) and added to the HA solution dropwise, followed by stirring for 24 h at room temperature. The resultant solution was dialyzed (MWCO: 3500) using 50% DMF for 24 h to remove excess ICG-NH_2_ and then dialyzed using deionized water for another 48 h to remove DMF. Finally, the solution was lyophilized to obtain the HA-ICG and stored at −20 °C for further use.

#### 2.2.3. Synthesis of MWNT-HA-ICG

MWNT-COOH (10 mg) and CDI (12 mg) were dissolved in 6 mL formamide and stirred for 1 h to activate the carboxyl groups of MWNT-COOH. Afterwards, the activated MWNT-COOH was dropped into HA-ICG solution in formamide with DMAP (15 mg) and stirred for 24 h under the protection of nitrogen. Next, the mixed solution was dialyzed (MWCO: 8000–14000) using 50% DMF for 24 h to remove the excess CDI and DMAP and then dialyzed using deionized water for another 48 h to remove the organic solvent. The final MWNT-HA-ICG was lyophilized and stored at −20 °C for further use.

#### 2.2.4. Synthesis of MWNT-HA-ICG/DOX

First, 10 mg MWNT-HA-ICG and 10 mg DOX·HCl were dissolved in deionized water, respectively. Then, DOX solution was added into MWNT-HA-ICG solution and stirred for 24 h at room temperature. Afterwards, the solution was centrifuged (12,000 rpm, 10 min), and the precipitate was washed with PBS (pH 7.4) to remove free DOX by repeated centrifugation. Finally, the resultant precipitate was MWNT-HA-ICG/DOX and was lyophilized and stored at −20 °C for further use.

### 2.3. Characterization of MWNT-Based Formulations

Solid samples of raw MWNT and MWNT-COOH were prepared to obtain their Raman spectra using confocal micro-Raman spectroscopy (LabRam HR800, Paris, France). The successful synthesis of HA-ICG and the grafting percentage of ICG on HA were confirmed by ^1^H-NMR spectroscopy (Avance™ 600, Bruker, Germany, 300 MHz), UV, and fluorescence spectroscopy. For the measurement of ^1^H-NMR spectra, HA and HA-ICG were dissolved in D_2_O, and ICG-NH_2_ was dissolved in DMSO-*d_6_*. Then, the measurement was carried out using 300 MHz under 20 °C. UV spectra were collected by dissolving ICG-NH_2_, DOX, MWNT-COOH, MWNT-HA-ICG, and MWNT-HA-ICG/DOX into deionized water, following by the scanning of their wavelength from 200–900 nm. The fluorescence spectra of samples were obtained by dissolving ICG-NH_2_, DOX, and MWNT-HA-ICG/DOX into deionized water, which was then irradiated by 760 nm and 490 nm excitation light to obtain their fluorescence spectra. In addition, thermogravimetric analysis (TGA) was performed to analyze raw MWNT, MWNT-COOH, and MWNT-HA-ICG (heat from 30 °C to 700 °C, nitrogen, heating rate at 10 °C/min). The particle size and zeta potential of various MWNT-based formulations were measured by dynamic light scattering (DLS) using a Malvern Zetasizer (Nano ZS-90, Malvern Instruments Ltd., Malvern, UK). The morphology of raw MWNT, MWNT-COOH, and MWNT-HA-ICG/DOX was verified by transmission electron microscope system (TEM, Hitachi, Japan, 80 kV). 

### 2.4. In Vitro Release of DOX from MWNT-HA-ICG/DOX

In vitro drug release profiles of DOX from MWNT-HA-ICG/DOX were performed at 37 °C in three different pH phosphate-buffered solutions (PBS). Briefly, 1 mg MWNT-HA-ICG/DOX was dispersed in 2 mL release medium with pH of 7.4, 6.5, or 5.5 in dialysis bag (MWCO: 3500). Then, the dialysis bag was placed in 40 mL corresponding pH release medium. The resultant dispersions were gently shaken at 37 °C in water bath at 100 rpm. At predetermined time intervals, 4 mL of release medium was taken out and the amount of released DOX was determined by a fluorospectrophotometer (HORIBA, Fluoromax-4, Palaiseau, France). In the meantime, 4 mL of same fresh release medium was replenished.

### 2.5. In Vitro Photothermal Effect of MWNT-Based Formulations

First, 1 mL of different concentrations free ICG-NH_2_, MWNT-COOH, and MWNT-HA-ICG/DOX (containing 20 μg/mL ICG-NH_2_ and 100 μg/mL MWNT-COOH) were dispersed in PBS and placed in 4 mL tubes. Then, different prepared solutions were irradiated by 808 nm laser (1 W/cm^2^) for 5 min. The temperatures were recorded every 25 s, and thermographic maps of the dispersion in tubes were taken by thermo imager (FLIR, E64501, Goleta, CA, USA) at the time point of 5 min.

### 2.6. In Vitro Cytotoxicity Assay

The cytotoxicity of various prepared formulations was evaluated through MTT assay. Briefly, MCF-7 cells were seeded in 96-well plates at a density of 1 × 10^4^ cells/well and incubated for 24 h. For treatment groups without laser irradiation, the culture medium was replaced with 200 μL medium containing ICG-NH_2_, DOX, MWNT-HA, MWNT-HA/DOX, MWNT-HA-ICG, and MWNT-HA-ICG/DOX at different concentrations and incubated for 24 h. For treatment groups with laser irradiation, the culture medium was replaced with 200 μL medium containing ICG-NH_2_, MWNT-HA/DOX, MWNT-HA-ICG, MWNT-HA-ICG/DOX, and MWNT-HA and incubated for 4 h, followed by laser irradiation (1 W/cm^2^, 5 min); then, laser treatment groups were further incubated for 20 h. After the total 24 h incubation of treatment groups with/without laser irradiation, 20 μL MTT solution (5 mg/mL) was added to each well, and the cells were incubated for 4 h. Afterwards, the medium was removed, and 150 μL DMSO was added. The absorbance measured by microplate reader (Thermo, Multiskan FC) at 570 nm. The cell viability was calculated using the following equation: Cell viability (%) = (A_sample_ − A_blank_)/(A_control_ − A_blank_) × 100%.

### 2.7. Cellular Uptake and Intracellular Trafficking

MCF-7 cells were seeded in 24-well plates and cultured until 80% cell confluence. The medium was discarded, and cells were washed twice with PBS before adding serum-free medium with ICG-NH_2_, DOX, MWNT-HA-ICG, MWNT-HA/DOX, and MWNT-HA-ICG/DOX at a final concentration of 10 μg/mL ICG-NH_2_ and/or 10 μg/mL DOX for cellular uptake for 1, 2, and 4 h. For competition assay, cells were pretreated with free HA (5 mg/mL) for 4 h before adding MWNT-HA-ICG/DOX. The cellular uptake of different formulations was determined by flow cytometry (BD FACS Calibur, San Jose, CA, USA) quantitatively according to the fluorescent property of ICG and DOX. The intracellular fluorescence of the above formulations was observed under a confocal laser scanning microscope (CLSM, Leica TCS SP5, Wetzlar, Germany) after staining cells with DAPI.

For intracellular trafficking study, cells were cultured, incubated with different formulations, washed, and fixed by 4% paraformaldehyde for 15 min. After discarding the paraformaldehyde, DAPI was added to stain the cell nucleus for 10 min. Finally, distribution of different formulations was observed by CLSM.

### 2.8. Cell Apoptosis Assessment

The in vitro antitumor efficacy was carried out by an apoptosis experiment. MCF-7 cells were seeded in 24-well plates at a density of 5 × 10^4^ cells/well and incubated for 24 h. Cells were co-incubated with control (PBS), free DOX, and MWNT-HA-ICG/DOX (containing 10 μg/mL ICG and 10 μg/mL DOX) for 24 h to evaluate the chemotherapeutic efficacy of constructed nanocomplexes. To study the photothermal therapeutic efficacy, cells were co-incubated with control (PBS), ICG-NH_2_, MWNT-HA-ICG, MWNT-HA/DOX, and MWNT-HA-ICG/DOX (containing 10 μg/mL ICG and 10 μg/mL DOX) for 4 h and then irradiated for 5 min (1 W/cm^2^), followed by another 20 h incubation. Afterwards, cells were collected and washed with PBS. Next, cells were suspended in binding buffer and stained with Annexin V-FITC/PI apoptosis detection kit in dark. Finally, the apoptotic cells were detected by flow cytometry.

### 2.9. In Vivo Imaging Study

Mice were randomly divided into three groups and injected with ICG-NH_2_, MWNT-HA-ICG, and MWNT-HA-ICG/DOX at a dose of 10 μg ICG-NH_2_/mouse via tail vein, respectively. Due to the fluorescent characteristic of ICG, the in vivo distribution and targeting efficiency could be evaluated using an in vivo imaging system (FX PRO, Kodak, Rochester, NY, USA) at predetermined time points. After 12 h post-injection, mice were sacrificed to obtain the tumor and major organs for ex vivo imaging quantitative analysis using the same imaging system. The temperature changes of tumors during the irradiation (5 min, 1 W/cm^2^) were monitored by a thermo-imager (FLIR, E64501) after 6 h post-injection at the time points of 0 min and 5 min.

### 2.10. In Vivo Antitumor Efficacy

The in vivo antitumor efficacy was evaluated using MCF-7 tumor bearing xenograft nude mice with average tumor volume around 100 mm^3^. All the mice were weighed and randomly divided into nine groups. Of the nine groups, four groups were administrated with PBS, DOX, ICG-NH_2_, and MWNT-HA/DOX intravenously without irradiation, while the rest groups were treated with PBS, ICG-NH_2_, MWNT-HA-ICG, MWNT-HA/DOX, and MWNT-HA-ICG/DOX with laser irradiation. All the formulations were administrated at a dose of 10 μg ICG-NH_2_/mouse and/or 0.5 mg/kg DOX per mouse every 2 days. For laser groups, tumors on mice were irradiated by 808 nm laser (1 W/cm^2^) for 5 min at 6 h after injection with different formulations. In the meantime, the tumor volumes and body weights of mice were recorded every 2 days until day 14.

### 2.11. Statistical Analysis

The data were expressed as mean ± S.D. from triplicate experiments conducted parallelly, unless otherwise noted. Statistical analysis was carried out through one-way analysis of variance (ANOVA) test for comparison of multiple groups. Statistical significance was regarded as * *p* < 0.05, ** *p* < 0.01, or *** *p* < 0.001.

## 3. Results

### 3.1. Synthesis of MWNT-HA-ICG/DOX

**Figure 1:**
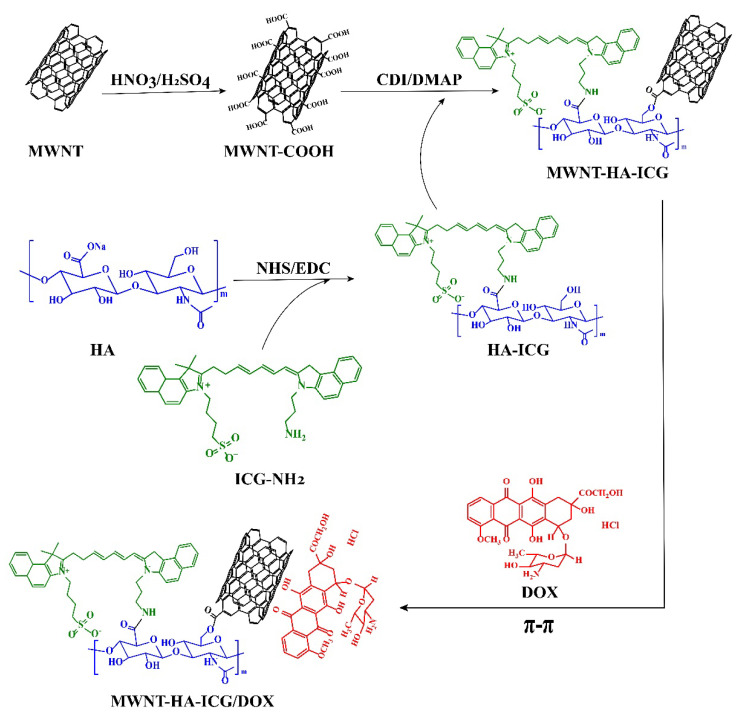
Synthesis route of MWNT-HA-ICG/DOX.

Pristine MWNT was long and covered by impurities, which hindered the direct application of MWNT as a drug delivery vehicle. Therefore, mixed acid (H_2_SO_4_/HNO_3_ *v*/*v* = 3:1) was used to remove the impurities, shorten the length of MWNT, and enable it to be modified with carboxyl group for further reaction. Moreover, MWNT is highly hydrophobic, which restricts its application in vivo. Thus, hydrophilic polymers are essential to be conjugated on the surface of MWNT to improve its dispersity. Hyaluronic acid (HA) is a negative polysaccharide with high molecular weight and consists of repeated D-glucuronic acid and *N*-acetyl-D-glucosamine disaccharide units [30]. HA has great water solubility and active hydroxyl and carboxyl groups, making it a prospective connection between MWNT-COOH and ICG-NH_2_ [31]. As the synthesis route shown in Figure 1, ICG-NH_2_ and HA were firstly conjugated through amide reaction, the resultant HA-ICG improved the water solubility of ICG-NH_2_ and was used as the reactant in the second step. Next, hydroxyl group on HA-ICG and carboxyl group on MWNT-COOH were connected through ester bond. By virtue of excellent water solubility of HA, the dispersity of MWNT-HA-ICG complex was greatly improved. Moreover, both the photosensitizer ICG and the drug vector MWNT have excellent optical features, which enabled ICG and MWNT to realize synergistic PTT effect. To further improve the antitumor efficacy, chemotherapy drug DOX was conjugated onto the wall of MWNT via a π–π bond; thus, the final MWNT-HA-ICG/DOX nanocomplexes could be obtained.

### 3.2. Characterization of MWNT-HA-ICG/DOX

**Figure 2:**
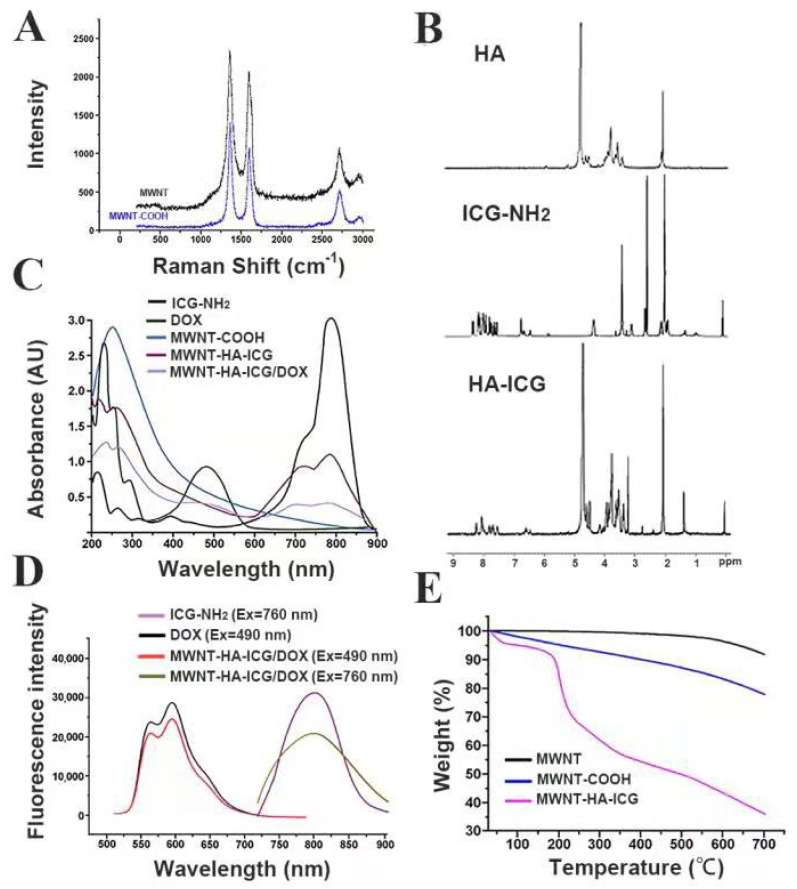
(**A**) Raman spectra of MWNT and MWNT-COOH. (**B**) ^1^H-NMR spectra of HA, ICG-NH_2_, and HA-ICG. (**C**) UV spectra of ICG-NH_2_, DOX, MWNT-COOH, MWNT-HA-ICG, and MWNT-HA-ICG/DOX. (**D**) Fluorescence spectra of ICG-NH_2_, DOX, and MWNT-HA-ICG/DOX. (**E**) TGA curves of MWNT, MWNT-COOH, and MWNT-HA-ICG.

The successful synthesis of MWNT-HA-ICG/DOX was characterized by Raman, ^1^H-NMR, UV, fluorescence spectra, and thermogravimetric analysis (TGA). As shown in Figure 2A, both Raman spectra of MWNT and MWNT-COOH displayed two characteristic peaks. Tangential G band (~1590 cm^−1^) represented the in-plane vibration of C–C bond, and the D band (~1350 cm^−1^) reflected the disorder in the carbon system. I_D_/I_G_ value of raw MWNT was 1.13, and the value of MWNT-COOH increased to 1.31. The increased value of I_D_/I_G_ could reflect the elevated degree of deficiency of MWNT, probably due to the carboxylation of MWNT. The conjugation between carboxyl groups on HA and amine groups of ICG-NH_2_ was verified by ^1^H-NMR. The solvent for HA and HA-ICG was D_2_O, and the solvent for ICG-NH_2_ was DMSO-*d*_6_. As shown in Figure 2B, the typical chemical shift of -CH- in HA was identified at 4.4~4.6 ppm, and the characteristic peaks of N-acetyl group and hydroxyl groups in HA were identified at 2.0 ppm and 4.0 ppm, respectively [32]. In addition, the evident peak of Ar-H due to proton resonance and the characteristic peak of -CH=CH- in ICG-NH_2_ were identified at 7.5–8.3 ppm and 6.3–6.7 ppm, respectively. In the ^1^H-NMR spectrum of HA-ICG, the characteristic peaks of HA and ICG-NH_2_ were both identified, manifesting the successful conjugation of ICG-NH_2_ onto HA. Moreover, as the UV spectrum was illustrated in Figure 2C, free ICG-NH_2_, DOX, and MWNT-COOH had absorbance peaks at 780 nm, 496 nm, and 253 nm, respectively, while modified MWNT-HA-ICG showed absorbance at wavelengths of 785 nm and 253 nm, and the final MWNT-HA-ICG/DOX displayed absorbance peaks at 785 nm, 496 nm, and 253 nm, indicating that HA-ICG was connected onto the wall of MWNT-COOH, and MWNT-HA-ICG/DOX was successfully synthesized. Similarly, as the fluorescence spectra shown in Figure 2D, free ICG-NH_2_ and DOX could be excited by wavelengths of 760 nm and 490 nm, which showed emission peaks at 803 nm and 590 nm, respectively. Meanwhile, MWNT-HA-ICG/DOX also had emission peaks at the wavelength of 792 nm and 590 nm, confirming the successful synthesis of MWNT-HA-ICG/DOX.

TGA is a common way to determine the contents of grafting substance on MWNT by measuring the weight loss from 30 °C to 700 °C. As shown in Figure 2E, pristine MWNT was quite stable when the temperature was below 500 °C. In the range of 500 °C to 700 °C, there was around 9.84% weight loss of MWNT, mainly due to the impurities on pristine MWNT. With the temperature rising, the carboxyl groups on MWNT-COOH decomposed. Additionally, at a temperature of 700 °C, the weight loss of MWNT-COOH was approximately 22.37%, which implied that the content of carboxyl groups in MWNT-COOH was about 12.53%. For MWNT-HA-ICG, its weight loss accompanied with temperature increment also reflected the amount of HA-ICG grafted on MWNT-COOH, which was approximately 43.25%, demonstrating that HA-ICG could be effectively conjugated on the wall of MWNT-COOH.

### 3.3. Particle Size, Zeta Potential, and Morphology

**Figure 3:**
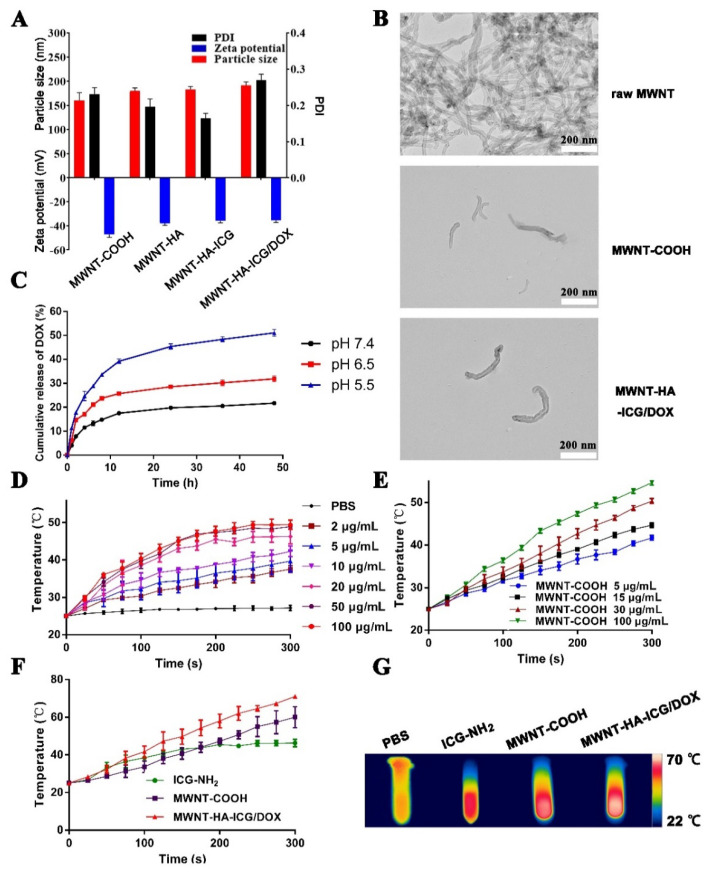
(**A**) Hydrodynamic diameter, zeta potential, and PDI of different MWNT-based formulations. (**B**) TEM images of raw MWNT, MWNT-COOH and MWNT-HA-ICG/DOX. (**C**) Cumulative release curves of DOX from MWNT-HA-ICG/DOX in three phosphate buffers with different pH. In vitro temperature curves of (**D**) free ICG-NH_2_, (**E**) MWNT-COOH at various concentrations, and (**F**) free ICG-NH_2_ (20 μg/mL), MWNT-COOH (100 μg/mL) and MWNT-HA-ICG/DOX after laser irradiation for 5 min (808 nm, 1.0 W/cm^2^). (**G**) Infrared thermographic maps of free ICG-NH_2_, MWNT-COOH, and MWNT-HA-ICG/DOX determined at 5 min after continuous laser irradiation.

The particle size, zeta potential, and polydispersity index (PDI) were measured to investigate the variation of MWNT after modified with different molecules. As is shown in Figure 3A, the particle size of MWNT-HA-ICG/DOX was around 190 nm and was larger than that of MWNT-COOH, which was due to the functionalization of ICG-conjugated HA and the non-covalent loading of DOX. In addition, the negative-charged nanocomplexes tended to be more stable in vivo due to their avoidance of the interaction with the negative-charged components in plasma [33,34]. The morphology of MWNT, MWNT-COOH, and MWNT-HA-ICG/DOX were observed by transmission electron microscopy (TEM). As shown in Figure 3B, raw MWNT was long, twined, and aggregated together with impurities on its surface. After acidification, MWNT-COOH was much shorter than raw MWNT, with a particle size of approximately 160 nm, which was in accordance with the result measured by dynamic light scattering (DLS). Moreover, the surface of MWNT-COOH was very smooth and no longer woven together, demonstrating that the mixed acid could efficiently shorten the raw MWNT and remove the impurities. After conjugation with HA-ICG and loading with DOX, the thickness of the MWNT was obviously increased and the length of functionalized MWNT was a little longer, demonstrating that HA-ICG and DOX were successfully attached to the surface of MWNT-COOH.

### 3.4. In Vitro Drug Release and Photothermal Effect of MWNT-Based Formulations

To investigate the in vitro drug release profile of DOX from MWNT-HA-ICG/DOX, three different pH phosphate buffers were used to simulate physiological pH, tumor pH, and lysosomal pH, respectively. As illustrated in Figure 3C, the cumulative released amount of DOX was pH-dependent. At pH 7.4, approximately 20% DOX was released from MWNT-HA-ICG/DOX after 48 h, implying that DOX would not release too much under normal physiological environment because the π–π stacking interaction between DOX and MWNT was stable. In contrast, approximately 30% and 50% of the loaded DOX was released from MWNT-HA-ICG/DOX at pH 6.5 and pH 5.5 after 48 h, respectively, suggesting the accelerated release profile of DOX in acidic tumor sites after internalization inside the tumor cells through receptor-mediated endocytosis. Altogether, the data above demonstrated that DOX could be released from MWNT-HA-ICG/DOX in a sustained manner in tumor sites and the constructed nanocomplexes were stable under normal physiological condition, which was attributed to attenuated π–π stacking interaction between DOX and MWNT due to the amino protonation of DOX under acidic PH conditions. To determine the in vitro PTT efficiency of ICG-NH_2_, MWNT-COOH, and the synergistic effect of ICG-NH_2_ and MWNT-COOH, a series of concentrations of the aforementioned three solutions were irradiated for 5 min (808 nm laser, 1.0 W/cm^2^) and the corresponding temperatures were recorded at the designed time points. As shown in Figure 3D,E, the photothermal performance of both free ICG-NH_2_ and MWNT-COOH exhibited a concentration-dependent and time-dependent profile. The temperature of MWNT-COOH was almost proportional to time, while the temperature of ICG-NH_2_ was not that case. When the concentration of ICG-NH_2_ was relatively low, the temperature increased significantly with the increment of its concentration and time. However, when the concentration of ICG-NH_2_ was over 50 μg/mL, the temperature did not show an obvious increase with the increment of concentration, which was probably due to the aggregation of ICG-NH_2_ that blunted its photothermal properties. As illustrated in Figure 3F, the temperature of MWNT-HA-ICG/DOX that contained 20 μg/mL ICG-NH_2_ and 100 μg/mL MWNT-COOH was higher than that of free ICG-NH_2_ and MWNT-COOH alone, demonstrating the synergistic photothermal effect of ICG-NH_2_ and MWNT-COOH. Moreover, as shown in Figure 3G, the thermographic maps taken at 5 min after laser irradiation displayed an increased temperature in MWNT-HA-ICG/DOX group compared with the others, which was in accordance with the results above.

### 3.5. In Vitro Cytotoxicity Studies

**Figure 4:**
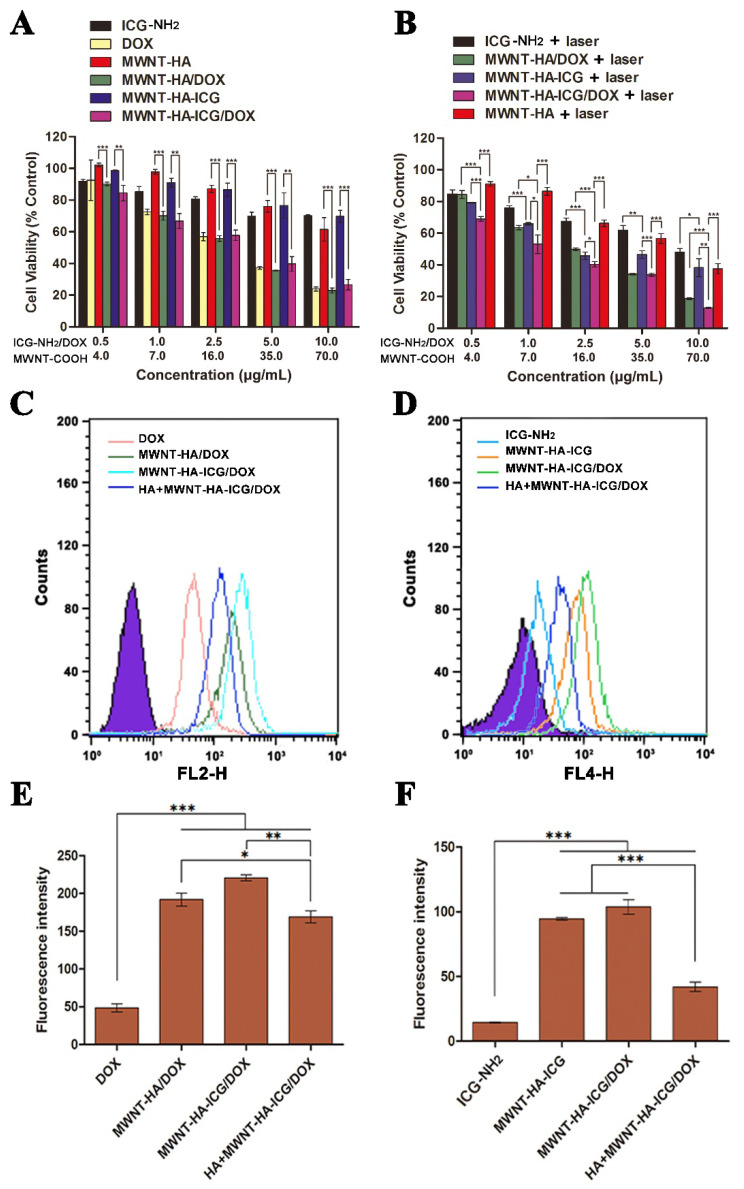
Cell viabilities of MCF-7 cells treated with different formulations at a series of concentrations of ICG-NH_2_/DOX and MWNT-COOH for 48 h (**A**) without laser irradiation and (**B**) with laser irradiation (ICG-NH_2_/DOX means ICG-NH_2_ and/or DOX). Flow cytometric profiles and fluorescence intensities of different formulations of (**C**,**E**) 10 μg/mL DOX, (**D**,**F**) 10 μg/mL ICG-NH_2_ at 4 h after treatment. Data were expressed as mean ± S.D. (*n* = 3). * p < 0.05, ** p < 0.01, and *** p < 0.001.

In vitro cell viability of free ICG-NH_2_, free DOX, and different MWNT-based formulations with or without laser irradiation was evaluated using MCF-7 cells by MTT assay. Since the amount of ICG-NH_2_ and DOX in constructed nanocomplexes was the same, and their concentrations were consistent with the concentration increment of MWNT-COOH, cell viability assays were carried out according to the concentrations of therapeutic agents (ICG-NH_2_/DOX and MWNT-COOH). Figure 4A showed the cell viability of different treatment groups without laser irradiation, which illustrated that the cell viability of free ICG-NH_2_ group was relatively high, implying the hypotoxicity of ICG-NH_2_. Meanwhile, the cell viability after MWNT-HA and MWNT-HA-ICG treatment decreased as the concentration increased, indicating that the cytotoxicity of MWNT-HA and MWNT-HA-ICG was concentration-dependent. However, the overall cytotoxicity of this nanocarrier was relatively low, even at the highest concentration, which was attributed to the improved biocompatibility and reduced cytotoxicity of MWNT through HA modification. Furthermore, the cytotoxicity of MWNT-HA/DOX and MWNT-HA-ICG/DOX was significantly higher than that of MWNT-HA and MWNT-HA-ICG, which was mainly due to the cytotoxic effect of DOX. For the laser treatment groups shown in Figure 4B, MCF-7 cell viabilities upon laser irradiation (808 nm, 1.0 W/cm^2^) for 5 min declined than those of unirradiated groups in Figure 4A. The higher the concentration of ICG-NH_2_ and MWNT-COOH, the better the photothermal therapeutic effect. In addition, from all the treatment groups in Figure 4B, cells incubated with MWNT-HA-ICG/DOX plus laser irradiation showed the lowest viability, indicating the synergistic therapeutic effect through chemo-phototherapy of the constructed nanocomplexes.

### 3.6. In Vitro Cellular Uptake Studies

The evaluation of in vitro cellular uptake of ICG-NH_2_, DOX, and different MWNT-based formulations was carried out using flow cytometry. ICG-NH_2_ and DOX were used as fluorescent probes to quantitatively indicate the amount of MWNT-HA-ICG/DOX internalized by MCF-7 cells. In this experiment, ICG-NH_2_, DOX, and their corresponding MWNT-based formulations were divided into two groups to detect their fluorescent intensity in two channels according to the emission wavelength. As shown in Figure 4C,E, the fluorescent intensity of free DOX was the lowest compared to other groups, while the fluorescent intensities of MWNT-HA/DOX and MWNT-HA-ICG/DOX were significantly stronger than free DOX. Moreover, after pretreatment with HA, the cellular uptake of MWNT-HA-ICG/DOX was significantly reduced, implying that HA-modified MWNT-based formulations could enter the cells through CD44 receptor-mediated endocytosis [32]. All the results above demonstrated that MWNT-based formulations were more likely to enter the cells due to the targeting property of HA. Moreover, the results of cellular uptake by ICG-NH_2_ fluorescence shown in Figure 4D,F also confirmed the same cellular uptake mechanism of MWNT-HA-ICG/DOX, which were in accordance with the results obtained in Figure 4C,E.

### 3.7. Intracellular Distribution and Cell Apoptosis Studies

**Figure 5:**
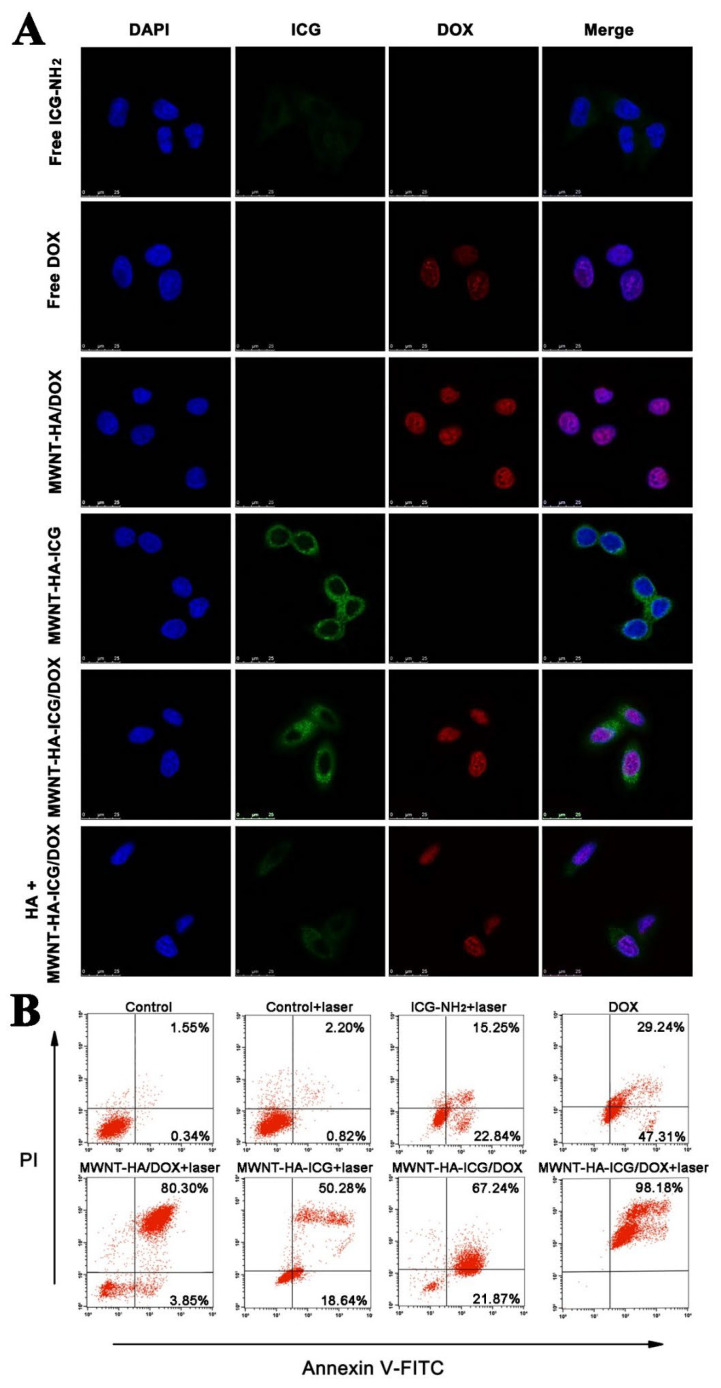
(**A**) Representative confocal images of intracellular trafficking of ICG-NH_2_, DOX, MWNT-HA/DOX, MWNT-HA-ICG, MWNT-HA-ICG/DOX, and MWNT-HA-ICG/DOX pretreated with HA in MCF-7 cells. Nucleus stained by DAPI showed blue fluorescence, ICG showed green fluorescence, and DOX showed red fluorescence. (**B**) Cell apoptosis induced by different formulations with and without laser irradiation using flow cytometry analysis. Cells treated with PBS were used as control.

CLSM was utilized to evaluate the intracellular distribution of MWNT-based formulations. DAPI was employed to dye the cell nucleus (blue). As shown in Figure 5A, the fluorescence of ICG-NH_2_ (green) and DOX (red) appeared in cytoplasm and nucleus, respectively. The merged fluorescence appeared light purple due to the fluorescent overlay of DOX (red) and DAPI (blue). More fluorescence of ICG-NH_2_ and DOX could be observed in MWNT-HA/DOX, MWNT-HA-ICG, and MWNT-HA-ICG/DOX groups, indicating that the functionalization of MWNT through HA enabled more ICG-NH_2_ and DOX to enter into tumor cells due to the targetability of HA. After pretreatment with HA, the cellular uptake of MWNT-HA-ICG/DOX obviously decreased, which was verified by the decreased ICG-NH_2_ and DOX fluorescence. In summary, the observation of CLSM and the results of flow cytometry were consistent. Cell apoptosis assay was carried out using flow cytometry to quantitatively evaluate the apoptosis-inducing efficacy of different formulations. The double staining of FITC labeled Annexin V and propidium iodide (PI) was used to discriminate live/early apoptotic cells and dead/late apoptotic cells [16]. As demonstrated in Figure 5B, only about 2% and 3% apoptotic cells were examined in the groups of control and control with laser irradiation, respectively, indicating that treatment using 808 nm laser was safe and would not cause damage to normal cells. In contrast, all the treatment groups using MWNT-based formulations exhibited obvious cell apoptosis. Notably, the combination therapy through MWNT-HA-ICG/DOX upon laser irradiation displayed the highest cell apoptotic rate of 98.18%, illustrating the synergistic chemo-photothermal therapeutic effect through the integration of DOX, ICG-NH_2_, and MWNT.

### 3.8. In Vivo Targeting Study

**Figure 6:**
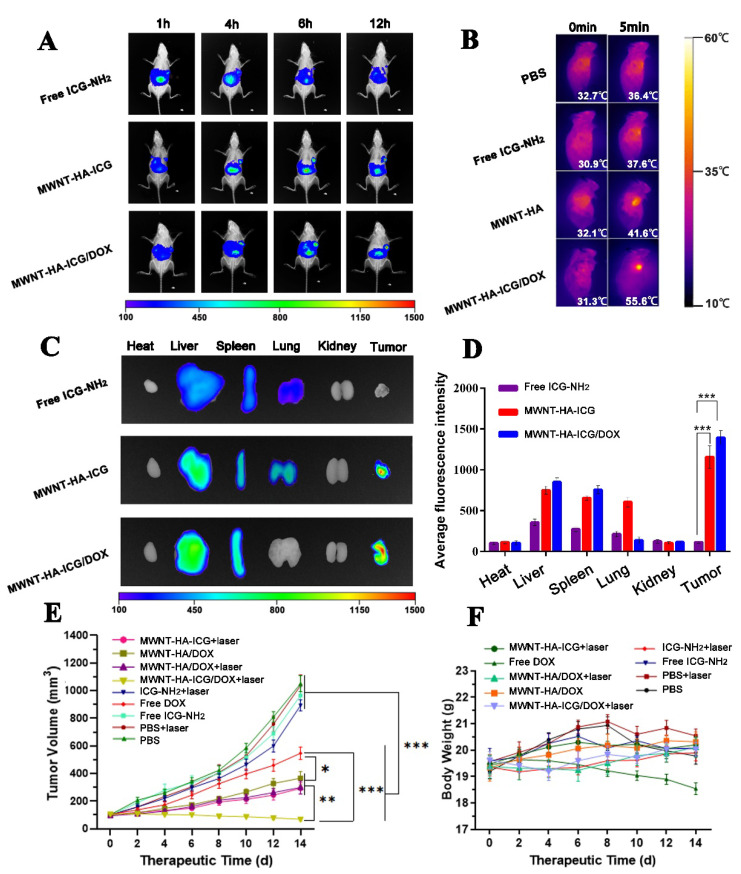
(**A**) Representative time-lapse in vivo imaging and biodistribution of MCF-7-tumor-bearing nude mice intravenously injected with ICG-NH_2_, MWNT-HA-ICG, and MWNT-HA-ICG/DOX. (**B**) Infrared thermographic maps of mice upon laser irradiation (808 nm, 1.0 W/cm^2^) at 6 h after IV injection with PBS, ICG-NH_2_, MWNT-HA, and MWNT-HA-ICG/DOX. (**C**) Representative ex vivo NIR imaging of tumors and major organs excised from mice at 12 h post-injection. (**D**) Quantitative analysis of the fluorescence intensity in tumors and major organs at 12 h post-injection. (**E**) Tumor growth and (**F**) body weight curves of mice after IV administered with different formulations. Data were expressed as mean ± S.D. (*n* = 3). * p < 0.05, ** p < 0.01 and *** p < 0.001.

A drug carrier that is able to target tumor sites plays a significant role in achieving elevated therapeutic outcome and reducing systemic side effects. Therefore, in vivo tumor targeting performance of constructed nanocomplexes were studied on MCF-7 xenograft tumor in nude mice using an in vivo fluorescence imaging system. As is shown in Figure 6A, mice were administered with ICG-NH_2_, MWNT-HA-ICG, and MWNT-HA-ICG/DOX and then photographed at the time points of 1 h, 4 h, 6 h, and 12 h. Mice injected with ICG-NH_2_ exhibited a primary liver accumulation with weak fluorescence at the tumor site. At 4 h post-injection, the fluorescence intensity of ICG-NH_2_ in the liver became weak, partly due to the quenching aggregation and instability of free ICG [35,36]. In contrast, mice administered with MWNT-based formulations exhibited strong fluorescent signal at the tumor sites, even at 12 h after injection, implying that nanocomplexes conjugated with HA were able to produce selective accumulation and retention at tumor sites due to the specific affinity of HA to overexpressed CD44 receptors on MCF-7 cells [37]. As illustrated in Figure 6C, after 12 h injection, tumors and major organs were isolated for ex vivo imaging, which demonstrated that ICG-NH_2_ was non-specifically accumulated in liver, spleen, and lung with no accumulation in tumor sites. In contrast, fluorescent images of mice treated with MWNT-HA-ICG and MWNT-HA-ICG/DOX showed favorable accumulation at tumor sites, which was attributed to the EPR effect and CD44-guided tumor targetability [38]. In the quantitative analysis shown in Figure 6D, the accumulation of nanocomplexes in tumor and major organs was consistent with that of ex vivo imaging above, which confirmed the excellent tumor targeting ability of the constructed nanocomplexes. In order to assess the PTT efficacy of the constructed formulations, the local temperatures of tumors upon laser irradiation (808 nm, 1.0 W/cm^2^) after 6 h treatment with PBS, free ICG-NH_2_, MWNT-HA, and MWNT-HA-ICG/DOX were recorded. As is illustrated in Figure 6B, the temperature of mice injected with ICG-NH_2_ increased from 30.9 °C to 37.6 °C after 5 min laser irradiation, which did not show apparent temperature enhancement compared to that in the mice injected with PBS solution, indicating that free ICG-NH_2_ could not specifically target tumor sites with a slow temperature increment. In contrast, due to the photothermal property of MWNT and targetability of HA, mice administered MWNT-HA showed an improved PTT performance over free ICG-NH_2_. Notably, the temperature of mice injected with MWNT-HA-ICG/DOX could rise to 55.6 °C after irradiation for 5 min, which was high enough for tumor ablation [39]. All the data above confirmed that the constructed nanocomplexes could contribute to a synergistic PTT effect due to the combination of MWNT and ICG-NH_2_ as well as the enhanced tumor targetability mediated by HA.

### 3.9. In Vivo Antitumor Efficacy Study

MCF-7 xenograft tumor model was established to investigate the synergistic antitumor efficacy of chemo-photothermal therapy during 14-day treatment. Mice were *i.v.* administered with different formulations with comparable amounts of therapeutics. As illustrated in Figure 6E, tumor volumes of mice treated with PBS, PBS plus laser, free ICG-NH_2_, and ICG-NH_2_ plus laser rapidly increased with the time, indicating that single treatment through PTT could not result in obvious inhibitory effect on tumor growth and ICG-NH_2_ plus laser treatment could not cause considerable antitumor effect either, which was mainly due to the lack of targetability towards tumor sites [40]. Meanwhile, mice injected with free DOX, MWNT-HA-ICG plus laser, MWNT-HA/DOX, and MWNT-HA/DOX plus laser showed a significantly slight tumor volume increment. Moreover, mice treated with MWNT-based formulations exhibited remarkably slower tumor growth compared to the free DOX group, which indicated that formulations based on MWNT-HA exhibited better tumor targetability, thus causing enhanced antitumor efficacy. In contrast, mice injected with the final MWNT-HA-ICG/DOX plus laser showed a significant tumor volume reduction compared to the other groups above, confirming the synergistic therapeutic efficacy of the chemo (DOX)-photothermal (MWNT and ICG-NH_2_) strategy in MWNT-HA-ICG/DOX nanocomplexes. Meanwhile, as Figure 6F illustrated, the body weight of mice treated with PBS and PBS plus laser increased in the first 8 days due to the growth of tumor and then decreased in the following days, which might be the result of the enlarged tumor that influenced the health of mice. In addition, the body weight of mice treated with DOX continuously reduced due to the cytotoxicity effect of DOX. Moreover, due to the non-specificity of DOX, treatment with free DOX also caused toxic effects on normal cells, which affected the life quality of mice and contributed to their weight reduction [41]. In contrast, the body weight of mice treated with MWNT-based formulations showed an increased tendency, indicating the reduced toxicity and increased therapeutic efficacy of constructed nanocomplexes that improved the life quality of mice, which was due to the elevated tumor-targeting ability and good biocompatibility of MWNT-based formulations after the modification of HA on MWNT.

## 4. Conclusions

In summary, we successfully fabricated a nano-based drug delivery system with synergistic PTT and chemotherapy effect for efficient tumor elimination. The integration of two photothermal agents ICG-NH_2_ and MWNT through the connection of HA could not only elevate the photothermal performance compared to the single treatment modality, but also improve the targetability of the whole nanocomplexes due to the specific binding of HA and CD44 receptors overexpressed in tumor cells. Moreover, a simultaneous therapeutic effect could be achieved after involving DOX in this drug delivery system. In vitro results showed that MWNT-HA-ICG/DOX plus laser irradiation could lead to significant cytotoxic effects towards MCF-7 cells. In vivo experiments demonstrated that the combinational treatment strategy through PTT and chemotherapy could result in a favorable inhibitory effect on MCF-7 tumor growth. Therefore, MWNT-HA-ICG/DOX provided a promising therapeutic opportunity based on synergistic strategies for cancer treatment.

## Data Availability

The datasets used and/or analyzed during the current study are available from the corresponding author on reasonable request.
